# Cloning, Expression, and Characterization of a New PL25 Family Ulvan Lyase from Marine Bacterium *Alteromonas* sp. A321

**DOI:** 10.3390/md17100568

**Published:** 2019-10-08

**Authors:** Jian Gao, Chunying Du, Yongzhou Chi, Siqi Zuo, Han Ye, Peng Wang

**Affiliations:** College of Food Science and Engineering, Ocean University of China, Qingdao 266003, China

**Keywords:** ulvan-derived oligosaccharides, ulvan lyase, heterologous expression, polysaccharide lyase family 25

## Abstract

Ulvan lyases can degrade ulvan to oligosaccharides with potent biological activity. A new ulvan lyase gene, *ALT3695*, was identified in *Alteromonas* sp. A321. Soluble expression of *ALT3695* was achieved in *Escherichia coli* BL21 (DE3). The 1314-bp gene encoded a protein with 437 amino acid residues. The amino acid sequence of ALT3695 exhibited low sequence identity with polysaccharide lyase family 25 (PL25) ulvan lyases from *Pseudoalteromonas* sp. PLSV (64.14% identity), *Alteromonas* sp. LOR (62.68% identity), and *Nonlabens ulvanivorans* PLR (57.37% identity). Recombinant ALT3695 was purified and the apparent molecular weight was about 53 kDa, which is different from that of other polysaccharide-degrading enzymes identified in *Alteromonas* sp. A321. ALT3695 exhibited maximal activity in 50 mM Tris-HCl buffer at pH 8.0 and 50 °C. ALT3695 was relatively thermostable, as 90% activity was observed after incubation at 40 °C for 3 h. The *K_m_* and *V_max_* values of ALT3695 towards ulvan were 0.43 mg·mL^−1^ and 0.11 μmol·min^−1^·mL^−1^, respectively. ESI-MS analysis showed that *enzymatic products* were mainly disaccharides and tetrasaccharides. This study reports a new PL25 family ulvan lyase, ALT3695, with properties that suggest its great potential for the preparation of ulvan oligosaccharides.

## 1. Introduction

Ulvan is a type of cell wall polysaccharide, extracted from marine green seaweed (genus *Ulva* and *Enteromorpha*) [1]. It is mainly composed of iduronic acid (IdoA), 3-sulfated rhamnose (Rha3S), xylose (Xyl), and glucuronic acid (GlcA) [1]. The repetitive disaccharide units in ulvan are Rha3S-GlcA, Rha3S-IdoA, and Rha3S-Xyl [1]. Ulvan has been shown to possess various pharmacological properties, such as antioxidant [2], anticoagulant [3], antitumor [4], antihyperlipidemic [5], and antiviral [6] activities. However, its biological activity is greatly affected by its molecular weight, and its application could be limited by its high viscosity [3,7,8]. Compared to high-molecular-weight ulvan, low-molecular-weight ulvan possesses higher solubility, lower viscosity, easier absorption, and greater exposure of reactive groups [3,5,9]. In addition, it has been reported that low-molecular-weight sulfated polysaccharides [7] and their iron complexes [10] have potent biological activity. Thus, ulvan-derived oligosaccharides have been attracting increased attention for their tremendous potential for applications in the functional food and pharmaceutical industries.

Several methods for depolymerizing polysaccharides have been studied. Qi et al. prepared different molecular weight ulvans by treatment with H_2_O_2_ and by changing the depolymerization conditions [7]. Yu et al. degraded ulvan by microwaving under different pressures [8]. Mild acid hydrolysis has also been used to degrade ulvan [1]. However, such chemical and physical methods have many shortcomings, including sulfate group loss, complex products, and low oligosaccharide yields [9]. In contrast, enzymatic depolymerization is a potentially energy-efficient and environmentally friendly method, with no requirement for harsh reagents or extreme conditions [11]. Moreover, specific enzymes can be very useful for studying polysaccharide structure [12,13]. Therefore, it is necessary to find new enzymes that are capable of degrading ulvan, such as ulvan lyases.

The first marine bacterium capable of degrading ulvan was isolated by Lahaye et al. [12]. However, no in-depth study of its ulvan-degrading enzymes has been reported. Recently, Collén et al. reported the first endolytic ulvan lyase genes [14], which were found in the ulvanolytic bacterium *Nonlabens ulvanivorans*. Subsequently, the genomes of three ulvanolytic bacteria were sequenced [15], and ulvan lyases belonging to different polysaccharide lyase families were found. Kopel et al. reported several ulvan lyases in these three genomes [15], and these ulvan lyases showed no homology to those found by Collén et al. [14], which belong to PL24 family. Foran et al. identified another novel ulvan lyase (LOR_29) in the *Alteromonas* sp. LOR genome, which is the founding member of polysaccharide lyase family 25 (PL25) [16]. Thus far, three ulvan lyase families have been established (http://www.cazy.org), including PL24, PL25, and PL28. Structural characterizations of representative enzymes from these three families have also been reported [17,18,19]. As the primary ulvan-degrading enzyme [16], ulvan lyase catalyzes β-elimination at the internal bond between uronic acid and Rha3S, producing oligosaccharides with unsaturated uronic acid (∆GlcA) [14,15]. Compared to other methods, the uniform enzymatic product is an advantage of using ulvan lyases to degrade ulvan, which is convenient for studying their pharmacological activity. In addition, sulfate groups are well retained during the degradation process, which is essential for the activity of ulvan oligosaccharides [9]. Ulvan lyases have also been used for epitope deletion studies [20]. However, only seven ulvan lyases have been characterized. To expand the repertoire of enzymes to efficiently produce ulvan-derived oligosaccharides, additional new ulvan lyases must be investigated.

Previous studies showed that *Alteromonas* sp. A321 was capable of degrading ulvan [9]. In this study, a new ulvan lyase gene, *ALT3695*, was identified in *Alteromonas* sp. A321 and soluble expression of ALT3695 was achieved in *Escherichia coli* BL21 (DE3). Recombinant ulvan lyases were purified and the molecular weight was investigated. ALT3695 differs from other enzymes previously found in *Alteromonas* sp. A321 [21]. Thus, this study reports a new enzyme for preparing ulvan-derived oligosaccharides and enriches the marine enzyme library.

## 2. Results and Discussion

### 2.1. Sequence Analysis

The *ALT3695* gene is 1314 bp in length and encodes a 437-amino acid protein. The ALT3695 amino acid sequence shares 64.14%, 62.68%, and 57.37% sequence identity with reported ulvan lyases from *Pseudoalteromonas* sp. PLSV (PLSV_3936, GenBank accession no. WP_033186995.1) [18], *Alteromonas* sp. LOR (LOR_29, GenBank accession no. WP_052010178.1) [16], and *Nonlabens ulvanivorans* PLR (NLR_492, GenBank accession no. WP_036580476.1) [16], respectively. As the representative enzyme of PL25 family, the structure and catalytic mechanism of PLSV_3936 have been investigated [18]. In PLSV_3936, His123, His143, Tyr 188, Arg204, and Tyr246 are conserved and have been proposed as active site residues, and Gln66, Tyr246, and Arg282 are highly conserved. 

**Figure 1:**
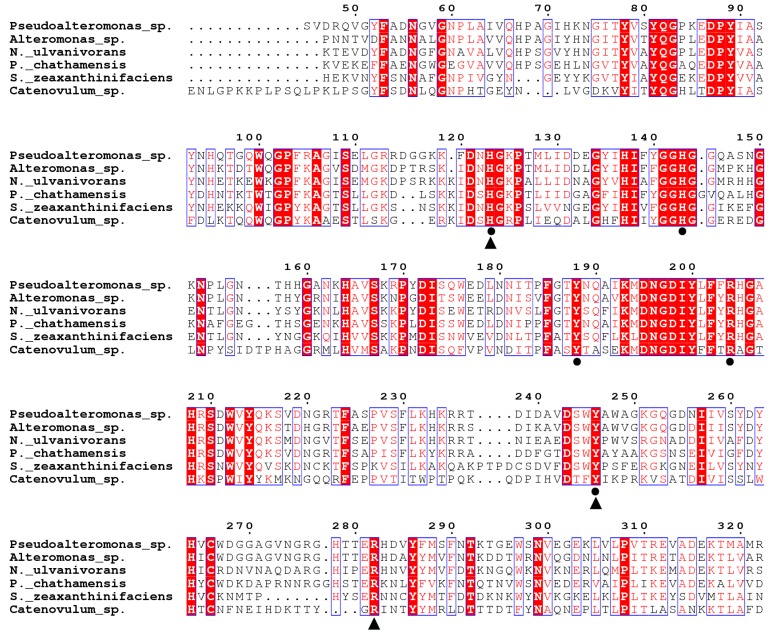
Amino acid sequence alignment of ALT3695 with ulvan lyases from *Pseudoalteromonas* sp. PLSV (PLSV_3936, GenBank accession no. WP_033186995.1), *Alteromonas* sp. A321 (ALT3695, GenBank accession no. MN347032), *Nonlabens ulvanivorans* PLR (NLR_492, GenBank accession no. WP_036580476.1), *Paraglaciecola chathamensis* S18K6 (GCHA_4617, GenBank accession no. GAC12534.1), *Siansivirga zeaxanthinifaciens* CC-SAMT-1 (AW14_13480, GenBank accession no. AJR04515.1), and *Catenovulum* sp. CCB-QB4 (C2869_03520, GenBank accession no. AWB65560.1). Active site residues are marked with filled circles (●). Highly conserved residues are marked with filled triangles (▲).

**Figure 2:**
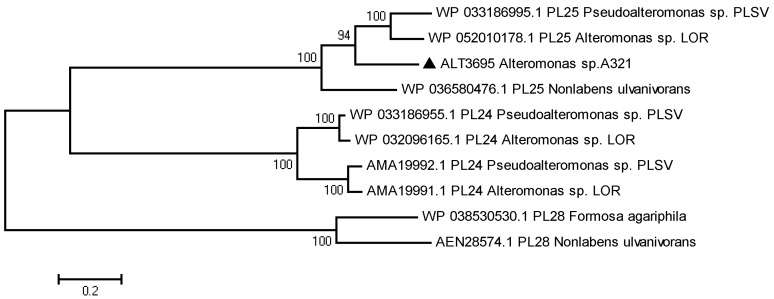
Phylogenetic tree of ALT3695 (filled triangle) and other ulvan lyases generated using the neighbor-joining method. Numbers along the branch nodes represent bootstrap percentages based on 1000 resamplings. The scale bar indicates the average number (0.2) of amino acid substitutions per site. *Pseudoalteromonas* sp. PLSV (PLSV_3936, GenBank accession no. WP_033186995.1), *Alteromonas* sp. LOR (LOR_29, GenBank accession no. WP_052010178.1), *Nonlabens ulvanivorans* (NLR_492, GenBank accession no. WP_036580476.1), *Pseudoalteromonas* sp. PLSV (PLSV_3925, GenBank accession no. WP_033186955.1), *Alteromonas* sp. LOR (LOR_107, GenBank accession no. AMA19991.1), *Pseudoalteromonas* sp. PLSV (PLSV_3875, GenBank accession no. AMA19992.1), *Alteromonas* sp. LOR (LOR_61, GenBank accession no. WP_032096165.1), *Formosa agariphila* KMM 3901 (BN863_22190, GenBank accession no. WP_038530530.1), and *Nonlabens ulvanivorans* PLR (IL45_01510, GenBank accession no. AEN28574.1).

Several homologous enzymes from different organisms with less sequence identity were selected in the carbohydrate-active enzymes (CAZy) database. Amino acid sequence alignment showed that most residues are also conserved in ALT3695 and other PL25 family members, except Gln66 (Figure 1). Among these residues, His143 and Try246 could help Arg204 to neutralize the negative charge on glucuronic acid. His123 and Tyr188 were acid-base catalysis residues [18]. A phylogenetic tree of ALT3695 and other reported ulvan lyases was constructed by the neighbor-joining method, which suggested that ALT3695 is a PL25 family ulvan lyase (Figure 2).

### 2.2. Expression and Purification of Recombinant ALT3695

**Figure 3:**
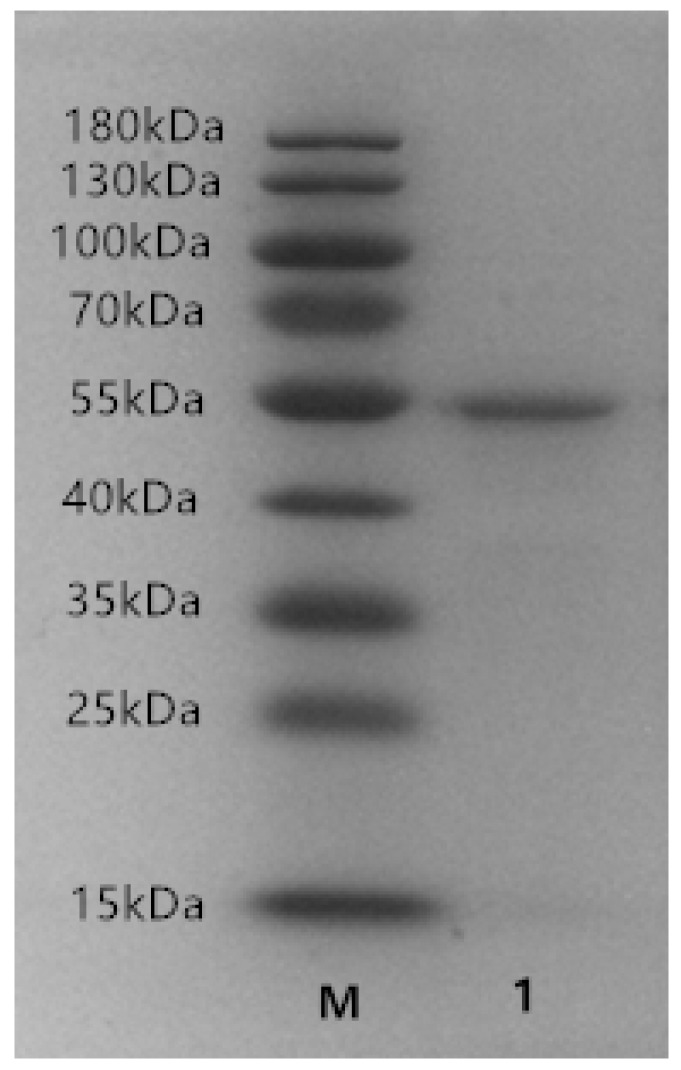
SDS-PAGE of purified ALT3695. Lane 1: purified ALT3695; Lane M: molecular weight marker.

Soluble expression of His-tagged ALT3695 ulvan lyase was achieved in *E. coli* BL21 (DE3) by adding 1 mM isopropyl-β-d-thiogalactopyranoside (IPTG). The recombinant ALT3695 was purified, and SDS-PAGE showed only a single protein band. The purified ALT3695 showed a specific activity of 1.88 U/mg protein, with 52% recovery (Table 1). As expected, the apparent molecular weight was 53 kDa (Figure 3), which is similar to that previously reported for PL25 family ulvan lyases, such as LOR_29 (52 kDa) [16] and NLR_492 (55 kDa) [16]. The molecular weight of ulvan lyases from other families, such as IL45_01510 (GenBank accession no. AEN28574.1, PL28) [14] and PsPL (GenBank accession no. AMA19992.1, PL24) [22], were about 46 kDa and 59 kDa, respectively.

### 2.3. Influence of Temperature on the Activity of Recombinant ALT3695

**Figure 4:**
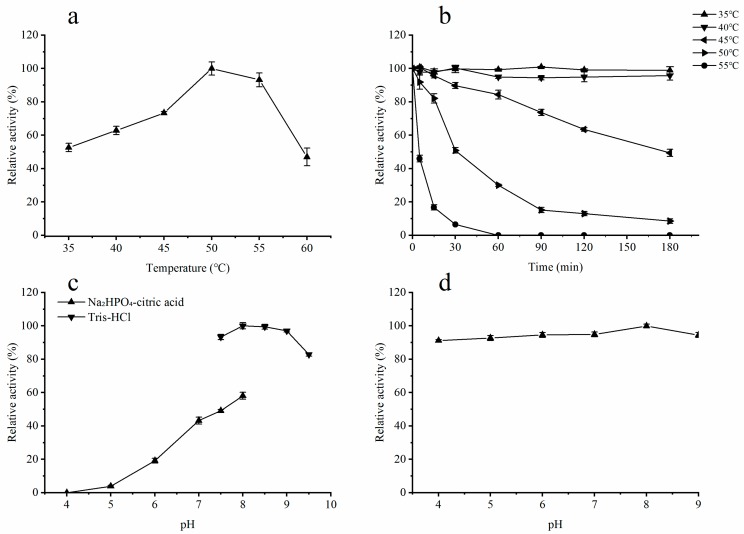
Biochemical characterization of ALT3695. (**a**) Activity of ALT3695 over a range of temperatures (35–60 °C) to determine the optimal temperature. (**b**) Thermal stability of ALT3695. (**c**) Activity of ALT3695 over a range of pH values to determine the optimal pH. (**d**) The pH stability of ALT3695.

The influence of temperature on enzyme stability and the optimum temperature for ALT3695 activity were investigated. Ulvan lyases from different families exhibit different optimum temperatures. The optimum temperature for ALT3695 activity was 50 °C (Figure 4a), which is higher than that of another ulvan lyase from the PL25 family (45 °C) [16]. Ulvan lyases from other families, such as FaPL28 (GenBank accession no. WP_038530530.1, PL28) [23] and PsPL (GenBank accession no. AMA19992.1, PL24) [22], showed maximum activity at lower temperatures of 29.5 °C and 35 °C, respectively. Little has been reported concerning the thermal stability of PL25 family members. FaPL28 had poor thermal stability, and less than 10% activity was observed after incubation for 3 h at or above 30.9 °C [23]. In contrast, ALT3695 was relatively thermostable and 90% activity was observed even after incubation for 3 h at 40 °C (Figure 4b). However, the stability of ALT3695 decreased as the temperature was increased above 40 °C (Figure 4b), 49% residual activity was observed after incubation for 3 h at 45 °C, and no activity was observed after incubation at 55 °C for 1 h.

### 2.4. Influence of pH on the Activity of Recombinant ALT3695

To investigate the effect of pH on ALT3695 activity, different buffers (pH 4–9.5) were used. The optimum pH of other ulvan lyases ranged from 7.5 to 9.0 [16,22,23], which corresponds to the pH of slightly alkaline seawater [24]. Similarly, ALT3695 showed maximal activity at pH 8.0 (Figure 4c). In addition, activity in Tris-HCl buffer was higher than that in Na_2_HPO_4_-citric acid buffer at pH 8.0, suggesting that the catalytic activity of ALT3695 was also affected by the buffer salt. ALT3695 retained 80% activity after pre-incubation at pH 4.0–9.0 for 2 h at 4°C, indicating that ALT3695 has excellent pH stability (Figure 4d).

### 2.5. Influences of Surfactants, Metal Ions, and Metal Chelators on the Activity of Recombinant ALT3695

Table 2a shows the influence of various metal ions on ALT3695 activity. K^+^ had no significant influence on ALT3695 activity. Additionally, Fe^2+^ and Cu^2+^ decreased enzyme activity, and this effect was also found in the ulvan lyases, PsPL [22] and FaPL28 [23]. Furthermore, ulvan lyases from different families exhibit different responses to Co^2+^, the activity of PsPL was increased by Co^2+^ [22], while the activity of FaPL28 [23] and ALT3695 was decreased by Co^2+^. This may be attributed to structural differences among these enzyme families. Moreover, Cd^2+^, Hg^2+^, and Fe^3+^ deactivated ALT3695 completely, which may be caused by the binding of these ions to the SH, CO, and NH moieties of amino acids, resulting in structural changes and inactivation [21]. It is worth mentioning that although ALT3695 has Zn^2+^-containing ligands, Zn^2+^ strongly inhibited enzymatic activity. Thus, Zn^2+^ may play a dual role, as inherent Zn^2+^ is essential for maintaining enzyme structure, but exogenous Zn^2+^ may bind to other amino acid residues and inhibit activity [25]. 

The enzyme activity was increased by Ca^2+^, Mg^2+^, and Ba^2+^. The addition of Ca^2+^ would not only increase the activity of PL25 ulvan lyase, but also stimulate the activity of PsPL [22] and FaPL28 [23]. With more Ca^2+^ added, the activity of ALT3695 was also increased. In addition, ALT3695 activity decreased by 68% after pre-incubation in 5 mM ethylenediaminetetraacetic acid (EDTA), similar phenomenon was also found in PsPL and FaPL28, this may due to the removal of divalent cations bound to enzymes. Additionally, structure analysis suggested that divalent metal ions play a structural role in all three ulvan lyase families.

The effect of surfactants and metal chelators on ALT3695 activity was also investigated (Table 2b). It has been reported that non-ionic surfactants could increase the activity of some enzymes [26,27]. However, surfactants had little effect on the activity of ALT3695. 

### 2.6. Kinetic Parameters of Recombinant ALT3695

*V_max_* is the maximum initial rate of an enzymatic reaction. Each enzyme has a specific *K_m_* for each substrate, which is inversely related to the enzyme’s affinity for the substrate [28]. The kinetic parameters of other ulvan lyases from the PL25 family have not yet been reported. The *K_m_* and *V_max_* of recombinant ALT3695 toward ulvan were 0.43 mg·mL^−1^ and 0.11 μmol·min^−1^·mL^−1^, respectively, as determined by the Lineweaver-Burk plot. ALT3695 showed a higher affinity for ulvan than PsPL and FaPL28, which had a *K_m_* of 2.10 mg·mL^−1^ [22] and 0.75 mg·mL^−1^ [23], respectively, suggesting the potential of ALT3695 for application.

### 2.7. Analysis of Enzymatic Products

The main repetitive disaccharide units in ulvan are Rha3S-GlcA, Rha3S-IduA, and Rha3S-Xyl. ALT3695 could cleave the bond on both Rha3S-GlcA and Rha3S-IdoA, while PL24 family ulvan lyase specifically cleaves the bond between Rha3S-GlcA. The products of ulvan degradation by ALT3695 were analyzed by negative electrospray ionisation mass spectrometry (ESI-MS). Mass spectroscopic analysis of products showed two major types of oligosaccharides. The peak at m/z 401 was equivalent to the disaccharide ∆GlcA-Rha3S, with a molecular weight of 402 Da. There was an unsaturated double bond between C4 and C5 [18]. The peak at m/z 379 was equivalent to double-charged molecular ions of the tetrasaccharide ∆GlcA-Rha3S-Xyl-Rha3S, with a molecular weight of 760 Da [14]. A low abundance of single-charged molecular ions of this tetrasaccharide was also identified as the peak at m/z 759. However, the products of ulvan degradation by PL24 ulvan lyase were different. The products included the two most abundant oligosaccharides which comprised of disaccharides (∆GlcA-Rha3S) and tetrasaccharides (∆GlcA-Rha3S-IdoA-Rha3S), with minor tetrasaccharides (∆GlcA-Rha3S-Xyl-Rha3S) [15].

**Figure 5:**
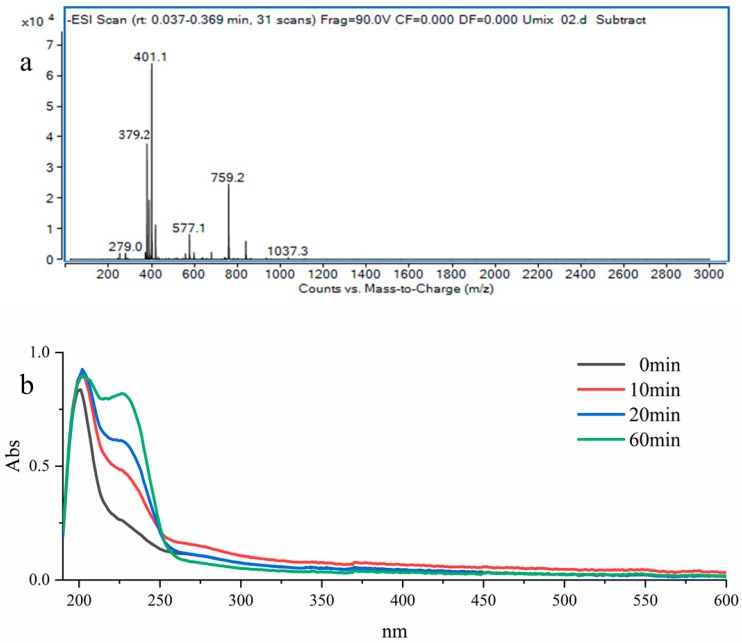
Analysis of the enzymatic products of ulvan generated by ALT3695. (**a**) ESI-MS analysis of products. (**b**) Full wavelength scans conducted during enzymatic degradation.

Moreover, the change in the full wavelength during enzymatic degradation was studied (Figure 5b). As the enzymatic reaction continued, the absorption peak at 235 nm gradually increased, which may be due to the generation of ∆GlcA-Rha3S and ∆GlcA-Rha3S-Xyl-Rha3S, as C-4=C-5 double bonds have a high absorbance at 235 nm [29].

## 3. Materials and Methods

### 3.1. Strains, Plasmids, and Medium

The ulvan-degrading strain *Alteromonas* sp. A321 was provided by Peng Wang (Ocean University of China, Qingdao, China). Genomic DNA was extracted, and the whole genome was sequenced. *E. coli* strains DH5α and BL21 (DE3) were purchased from TIANGEN Biotech Co. (Beijing, China). The pProEX-HTa vector was used to clone and express the ulvan lyase gene. The *E. coli* strains were cultured in Luria-Bertani medium.

### 3.2. Sequence Analysis

An ulvan lyase gene, named *ALT3695*, was identified in the genome, and its sequence was deposited into the NCBI database (GenBank accession no. MN347032). DNAMAN 9 (Lynnon Biosoft, San Ramon, CA, USA) software was used to analyze the sequence of *ALT3695*. The homology was analyzed using BLAST at the NCBI website. A phylogenetic tree based on the homology of various ulvan lyases was constructed using MEGA 5.0 (Koichiro, Tokyo, Japan).

### 3.3. Construction of the Recombinant ALT3695-Expressing Strain

The *ALT3695* gene was amplified by PCR using Phanta^®^ Turbo Super-Fidelity DNA Polymerase (Vazyme Biotech, Nanjing, China). Two primers were designed to amplify the *ALT3695* gene, 5′-CCGGAATTCCGGCCCAATAATACTGTCGACTTCGCTAACA-3′ and 5′-GGGGTACCCCTTACTTATTCTCGCTTCGTATTGGTCC-3′. Then, the amplicon was purified, digested, and ligated into the pProEX-HTa vector to generate pProEX-HTa-ALT3695. The recombinant plasmid pProEX-HTa-ALT3695 was transformed into *E. coli* DH5α for amplification, and then transformed into *E. coli* BL21(DE3) for expression. Positive strains were verified by sequencing.

### 3.4. Expression of Recombinant ALT3695

*E. coli* BL21 (DE3) cells containing pProEX-HTa-ALT3695 were cultivated in Luria-Bertani medium supplemented with ampicillin (100 μg/mL). The culture conditions were 37 °C and 200 rpm. When the strain entered logarithmic growth phase (OD600 ~ 0.7), IPTG was added at a final concentration of 1 mM to induce the expression of ALT3695. Then, the culture temperature was decreased to 28 °C, but 200 rpm was retained with further cultivation for 18 h. Finally, cells were collected by centrifugation under freezing conditions (4 °C) and resuspended in 20 mM Tris-HCl buffer (pH 7.5).

### 3.5. Enzyme Purification

After homogenization by sonication, the supernatant containing crude enzymes was obtained by centrifugation under freezing conditions. The His-tagged ulvan lyase was purified using BeaverBeads™ IDA-Nickel (Beaverbio, Suzhou, China) and eluted using 20 mM Tris-HCl buffer (pH 7.5) supplemented with 0.5 M NaCl and 300 mM imidazole. The concentration of soluble protein was determined by the Folin-Ciocalteu method [30]. Purified enzyme was then subjected to SDS-PAGE to analyze the purity and molecular mass [31].

### 3.6. Enzyme Activity Assay

Ulvan was extracted from *Ulva clathrate* (Fujian, China) according to the method of Qi et al. [32]. The ulvan substrate was added at 0.1% (w/v) in 50 mM Tris-HCl buffer (pH 8.0) and the reaction was conducted at 35 °C. The activity of ulvan lyase was examined by measuring the change in absorbance at 235 nm [14]. One unit (U) of ulvan lyase activity was defined as the amount of protein needed to generate 1 μmol of unsaturated glucuronyl residue (extinction coefficient, 4800 M^−1^·cm^−1^) per minute [23]. All assays were repeated three times.

### 3.7. Characterization of Recombinant ALT3695

To examine the optimum temperature of recombinant ALT3695, the reaction was conducted at 35–60 °C under standard conditions. The change in enzyme stability at different temperatures was analyzed by conducting enzyme activity assays after pre-incubating the enzyme for various times (5, 15, 30, 60, 90, 120, and 180 min) at 35–60 °C.

The optimal pH of recombinant ALT3695 was investigated by examining its activity in 50 mM Na_2_HPO_4_-citric acid buffer (for pH 4.0–8.0) and 50 mM Tris-HCl buffer (for pH 7.5–9.5). To investigate the change of enzyme stability by pH, purified enzymes were stored for 2 h at 4 °C in buffers of different pH levels, and then assayed for residual enzyme activity.

To examine the influence of different metal ions on recombinant ALT3695 activity, the enzyme was incubated with various metal ions (at 10 mM and 20 mM) for 2 h at 4 °C. The following metal ions were tested: K^+^, Ca^2+^, Mg^2+^, Zn^2+^, Ba^2+^, Cu^2+^, Fe^2+^, Hg^2+^, Co^2+^, Cd^2+^, and Fe^3+^. The effect of the following surfactants and metal chelators on recombinant ALT3695 activity was also determined: Tween-20, Tween-80, Triton X-100, EDTA, and 1,10-phenanthroline. The enzyme solution was stored with each surfactant or potential inhibitor (at 5 mM and 10 mM) for 2 h at 4 °C. Then, enzyme activity was assayed and compared with the activity in the absence of additive.

### 3.8. Kinetic Measurements

To determine the kinetic parameters of recombinant ALT3695 using ulvan as a substrate, reactions were performed with various concentrations (0.03125–1.0 mg·mL^−1^) of substrate under standard conditions for 5 min. Then, the *K_m_* and *V_max_* values were calculated by constructing a Lineweaver-Burk double reciprocal plot.

### 3.9. Analysis of Enzymatic Products

To obtain enzymatic products for analysis, purified ALT3695 was incubated with ulvan (5 mg·mL^−1^) for 3 h at 35 °C. Then, product samples were collected, and the product molecular weights were measured by negative-ion ESI-MS (Agilent 1290 Infinity II-6460, Agilent Corp., Wilmington, DE, USA; Frag = 90.0 V, m/z 100–3000 amu). Full wavelength scans (190–600 nm) were obtained during enzymatic degradation.

## 4. Conclusions

ALT3695 is a new ulvan lyase identified from *Alteromonas* sp. A321. The gene was cloned, and the recombinant ALT3695 was expressed in soluble fraction. The enzymatic properties were also investigated. The enzyme exhibited excellent stability and substrate affinity. Maximum activity was observed at 50 °C, and 90% activity remained after incubation at 40 °C for 3 h. ALT3695 catalyzes β-elimination at the internal bonds between uronic acids and Rha3S, leading to increased absorbance at 235 nm. ESI-MS results showed that disaccharides and tetrasaccharides were the major enzymatic products. In conclusion, ALT3695 shows great potential for the preparation of ulvan oligosaccharides. Further research on this recombinant strain and a structural analysis of ulvan lyase ALT3695 are warranted based on results of this study.

## Figures and Tables

**Table 1:** Characterization of purified ALT3695.

Purification Steps	Total Protein (mg)	Total Activity (U)	Specific Activity (U/mg)	Purification (fold)	Yield (%)
Crude enzymes	21.39	20.49	0.96	1.00	100.00
BeaverBeads™ IDA-Nickel	5.69	10.68	1.88	1.96	52.12

**Table 2:** (**a**) Effects of various metal ions on the activity of ALT3695 ulvan lyase. (**b**) Effects of surfactants and metal chelators on enzyme activity.

(**a**)				
**Metal Ions**	**Concentration (mM)**	**Relative Activity (%)**	**Concentration (mM)**	**Relative Activity (%)**
Control	-	100.01 ± 1.37	-	100.01 ± 1.37
K^+^	10	101.85 ± 1.66	20	103.62 ± 1.92
Ca^2+^	10	112.34 ± 0.48	20	142.51 ± 2.62
Mg^2+^	10	106.13 ± 0.42	20	117.13 ± 2.12
Zn^2+^	10	ND	20	ND
Ba^2+^	10	105.49 ± 2.03	20	121.23 ± 2.39
Cu^2+^	10	17.05 ± 0.68	20	ND
Fe^2+^	10	19.65 ± 2.85	20	ND
Co^2+^	10	29.88 ± 2.51	20	ND
Cd^2+^	10	ND	20	ND
Hg^2+^	10	ND	20	ND
Fe^3+^	10	ND	20	ND
(**b**)				
**Reagents**	**Concentration (mM)**	**Relative Activity (%)**	**Concentration (mM)**	**Relative Activity (%)**
Control	-	100 ± 0.21	-	100 ± 0.21
Tween-20	5	98.73 ± 2.22	10	84.17 ± 1.52
Tween-80	5	103.29 ± 0.44	10	101.14 ± 1.67
Triton X-100	5	104.01 ± 0.32	10	97.36 ± 1.85
EDTA	5	32.11 ± 1.23	10	ND
1,10-phenanthroline	5	106.71 ± 0.79	10	117.08 ± 1.71

ND: No activity was determined.
